# Stroke Severity in Ischemic Stroke Patients with a History of Diastolic Blood Pressure Treated in a Telestroke Network

**DOI:** 10.3390/jcdd9100345

**Published:** 2022-10-10

**Authors:** Christina Brown, Kameron Terrell, Richard Goodwin, Thomas Nathaniel

**Affiliations:** 1 Department of Biology, College of Charleston, Charleston, SC 29424, USA; 2 School of Medicine Greenville, University of South Carolina, Greenville, SC 29605, USA

**Keywords:** ischemic stroke, diastolic blood pressure, telestroke, stroke severity

## Abstract

Background: The relationship between diastolic blood pressure (DBP), risk factors, and stroke severity in acute ischemic stroke (AIS) patients treated in a telestroke network is not fully understood. The present study aims to determine the effect of risk factors on stroke severity in AIS patients with a history of elevated DBP. Material and Methods: We retrospectively analyzed data on stroke severity for AIS patients treated between January 2014 and June 2016 treated in the PRISMA Health telestroke network. Data on the severity of stroke on admission were evaluated using NIHSS scores ≤7 for reduced, and >7 for increased, stroke severity. DBP was stratified as ≤80 mmHg for reduced DBP and >80 mmHg for elevated DBP. The study’s primary outcomes were risk factors associated with improving neurologic functions or reduced stroke severity and deteriorating neurologic functions or increased stroke severity. The associations between risk factors and stroke severity for AIS with elevated DBP were determined using multi-level logistic and regression models. Results: In the adjusted analysis, AIS patients with a DBP ≤ 80 mmHg, obesity (OR = 0.388, 95% Cl, 0.182–0.828, *p* = 0.014) was associated with reduced stroke severity, while an increased heart rate (OR = 1.025, 95% Cl, 1.001–1.050, *p* = 0.042) was associated with higher stroke severity. For AIS patients with a DBP > 80 mmHg, hypertension (OR = 3.453, 95% Cl, 1.137–10.491, *p* = 0.029), history of smoking (OR = 2.55, 95% Cl, 1.06–6.132, *p* = 0.037), and heart rate (OR = 1.036, 95% Cl, 1.009–1.064, *p* = 0.009) were associated with higher stroke severity. Caucasians (OR = 0.294, 95% Cl, 0.090–0.964, *p* = 0.002) and obesity (OR = 0.455, 95% Cl, 0.207–1.002, *p* = 0.05) were more likely to be associated with reduced stroke severity. Conclusions: Our findings reveal specific risk factors that can be managed to improve the care of AIS patients with elevated DBP treated in the telestroke network.

## 1. Introduction

According to the American Heart Association guidelines [1], high blood pressure or hypertension is defined by two levels: (i) elevated blood pressure (BP), with a systolic pressure (SBP) between 120–129 mm Hg and diastolic pressure (DBP) less than 80 mm Hg, and (ii) with an SBP of 130–139 mm Hg and a DBP of 80–89 mm Hg. More than three-quarters of acute ischemic stroke (AIS) patients present with elevated BP when diagnosed. Of that three-quarters, half already have a history of hypertension. Some studies also linked BP in the acute phase of the stroke to poor outcomes [2].

While DBP between 70 and 80 mm Hg may be an appropriate indicator for lower stroke risk [2], elevated DBP > 80 mm HG indicates stroke risk [3]. Moreover, numerous studies have strongly linked a DBP > 80 mm Hg with hypertension [4,5]. Some clinical trials have revealed the relationship between hypertension and increased risk for AIS, but the relationship between BP at admission for AIS and related severity, including outcome, is controversial [6,7]. Some studies reveal poor outcomes in patients with elevated BP during the acute phase of stroke [2,8,9]. On the other hand, some other studies did not identify any association [10,11], whereas others suggested a U-shaped relationship [12]. More importantly, there are limited and conflicting results regarding whether SBP or DBP at admission is a better predictor of stroke severity. In some studies, neither SBP nor DBP predicted stroke severity [11,13], whereas, in others, only SBP [8,12] or DBP [3,14] was associated with worse neurologic outcomes. Recent clinical trials have mainly focused on SDP in reducing vascular risk [3,15]. However, little is known about the relationship between DBP level with severity after a stroke. The few studies that investigated the effect of elevated DBP on the severity of stroke focused on the non-telestroke setting [14,16]. Similar studies are yet to be implemented in the telestroke network. Therefore, the relationship between telestroke technology, stroke severity, and specific risk factors contributing to stroke severity is not fully understood. In addition, most of the existing studies on DBP did not separately analyze risk factors that contribute to stroke severity in AIS patients with elevated DBP > 80 mm Hg and those with DBP ≤ 80 mm Hg. Therefore, more data are needed to evaluate the relationship between DBP in the acute phase of stroke and stroke severity and to clarify whether elevated DBP alone or in combination with other risk factors are associated with higher stroke severity in AIS patients treated in the telestroke network.

The telestroke network provides time-effective treatments to patients in rural communities who may otherwise not have a stroke expert available [6,17,18]. In addition, it provides the necessary opportunity for medically underserved communities to obtain the appropriate care that matches current clinical practice [16,19,20]. Therefore, telestroke provides access to vascular neurology expertise for hospitals lacking stroke coverage and offers the technology to provide ongoing support to their patients [18,19,21]. A retrospective data analysis of specific factors contributing to stroke severity among AIS patients with elevated DBP in the telestroke network is an important step in identifying comorbidities that can be managed to improve stroke care for AIS patients in the telestroke network.

Several risk factors, such as diabetes, hypertension, atrial fibrillation, coronary and peripheral artery disease, heart failure, and age, have been associated with stroke severity [22,23,24]. In addition, the severity conferred by the different risk factors often clusters among those with pre-existing hypertension and may significantly contribute to stroke severity [25]. Therefore, risk factors associated with stroke severity in AIS with elevated DBP > 80 mm Hg may differ from those with DBP ≤ 80 mm Hg. Since there is a greater likelihood of stroke severity in AIS populations with elevated DBP > 80 mm Hg, we tested the hypothesis that more risk factors may contribute to stroke severity in AIS patients with elevated DBP > 80 mm Hg compared with those with DBP ≤ 80 mm Hg in the telestroke network. The goal is to understand how DBP interacts with other risk factors to increase stroke severity in the telestroke. The findings from this study may provide further insight into the understanding of risk factors associated with stroke severity in AIS patients with elevated DBP.

## 2. Methods

This is a retrospective data analysis of stroke data with a history of elevated DBP between January 2014 and June 2016 treated in the PRISMA Health telestroke network. Data for the present study were extracted from the electronic medical record from the PRISMA Health Stroke Registry. The stroke registry provides data for telestroke patients with a primary diagnosis of ischemic stroke and has been described in our previous studies [9,17]. Data for the patient demographics and clinical variables from telestroke patients were abstracted by a stroke nurse. In addition, all data were examined under quality control checks using an established protocol to regulate the quality of the data and prevent several types of errors, including errors in interpretation or coding and data entry.

For each patient, we collected data on basic demographic information (age, sex, race/ethnicity) and comorbidities, including atrial fibrillation/atrial flutter, coronary artery disease (CAD), and carotid stenosis. Other factors included depression, diabetes, drug or alcohol abuse, dyslipidemia, a family history of stroke, congestive heart failure (CHF), hormonal replacement therapy, hypertension, and migraine. We also collected data on obesity, prior stroke, prior TIA, prosthetic, peripheral vascular disease (PVD), chronic renal disease, sleep apnea, and history of smoking. Data were also collected from the National Institutes of Health Stroke Scale (NIHSS) score on arrival and history before stroke or transient ischemic attack, length of inpatient stay, and hospital discharge status. Inclusion criteria included ischemic stroke patients with clearly defined onset, a measurable deficit on the NIHSS, and a baseline brain CT scan without evidence of intracranial hemorrhage. Patients with intracranial hemorrhage, symptoms suggestive of subarachnoid hemorrhage, and seizure at onset of the stroke were excluded. In addition, we excluded data from AIS patients with no record of BP data and those not treated in the telestroke network. The description of hypertension is based on the guidelines for the management of hypertension pre and post-stroke. According to Joint National Committee (JNC7) guideline, stroke with hypertension (stages 1; SBP; 140–159, DBP 90–99 and stage 2; SBP > 160, DBP; or  >100) should be managed to levels or 130/80 mmHg or lower [26]. We focused on AIS patients with DBP ≤ 80 or >80 mm Hg at 24–72 h post-AIS, which is reported to be independently associated with favorable or poor outcomes in AIS patients with thrombolytic therapy [27]. Data were also collected on the mode of emergency department (ED) arrival, symptom onset time, and admission to ED. We collected data from patients directly admitted to the ED medical services (EMS) and those with indirect admission by being transferred to the ED in the telestroke network. In this study, onset time refers to the time the patient first presented with a neurological condition or the last normal observation for unknown clinical conditions. Laboratory analysis information was collected, including total cholesterol, triglycerides, HDL, LDL, lipids, blood glucose, and creatine. We collected data on the rate of ambulation at discharge, defined as the proportion of patients who ambulated independently or with assistance from another person among all stroke survivors. This study protocol was reviewed and approved by the Institutional Review Board of the PRISMA Health Institutional Committee for Ethics [approval #: 00052571].

### Statistical Analysis

All statistical analyses were performed utilizing SPSS Statistics Software version 26.0 (Chicago, IL, USA), and *p* < 0.05 was used to establish statistical significance in all comparisons between groups. A univariate analysis was used to determine risk factors associated with DBP ≤ 80 mm Hg or DBP > 80 mm Hg stratified by an NIHSS ≤ 7 or NIHSS > 7. We used descriptive statistics to determine AIS patients with elevated DBP risk factors. Continuous variables were analyzed using a Student’s *t*-test, while discrete variables were analyzed using Pearson’s Chi-squared test. Results were represented as percentages, and comparisons between groups were determined. We used the backward stepwise logistic regression to determine the odds ratios (ORs) and the corresponding 95% confidence intervals (CIs) for the primary outcome. In addition, an adjusted analysis was performed for the risk factors (e.g., age, sex, diabetes mellitus, dyslipidemia, atrial fibrillation, smoking). Therefore, the adjusted models included selected covariates depending on whether they were confirmed confounders in the bivariate analysis. Variables that were not significant were sequentially eliminated from the final model. 

The main considerations for our power analysis are related to the ability to detect differences between ischemic stroke patients with a history of ≤80 mm Hg and >80 mm HG Hg and NIHSS ≤ 7 and >7. The PASS version 16 was used to estimate the power analysis for 213 AIS with diastolic blood pressure > 80 mmHg and 239 diastolic blood pressure ≤ 80 mmHg. Our sample size of 239 for the ≤80 mm Hg and 213 for the >80 mm HG Hg AIs patients both yielded less than 0.6 power. For the NIHSS ≤ 7 and >7 categories, the power was 0.51. 

A prespecified subgroup analysis for risk factors and stroke severity effect on AIS-DBP patients was performed, with subgroups defined as AIS-DBP > 80 mmHg (stroke severity; NIHSS ≤ 7 and >7 groups), and AIS-DBP ≤ 80 mmHg (stroke severity; NIHSS ≤ 7 and >7). The logistic regression model used DBP categories (DBP > 80 mmHg or DBP ≤ 80 mmHg) and stroke severity groups as the dependent variable. In contrast, demographic and risk factors for the DBP > 80 mmHg or DBP ≤ 80 mmHg AIS patients were included in the model as primary independent variables. The primary outcome is the adjusted variables associated with stroke severity and worsening or improving neurologic functions. The final models’ ORs and 95% CI were estimated using conditional likelihood. The final adjusted models were assessed for multicollinearity. Interactions were checked among independent variables using the Hosmer–Lemeshow test. The area under the receiver operating curve (AUROC) for score prediction was used to determine the model’s sensitivity, specificity, and accuracy. 

## 3. Results

A total of 452 AIS patients were identified in this study. Of these, 239 patients presented with a DBP ≤ 80 mmHg, while 213 patients presented a DBP > 80 mmHg (Table 1). As shown in Table 1, AIS patients that presented with elevated DBP of >80 were less likely to be female (46.0% vs. 56.1%), present with coronary artery disease (27.2% vs. 37.2%), and dyslipidemia (47.9% vs. 53.6%). In addition, they were more likely to present with a higher heart rate (81.43 ± 15.99 bpm vs. 75.62 ± 15.25 bpm) and SBP (157.33 ± 22.1 mmHg vs. 137.24 ± 22.92 mmHg). 

Table 2 presents the demographic and clinical risk factors in AIS patients stratified by NIHSS scores (≤7 or >7) for AIS with DBP ≤ 80 mm Hg and >80 mmHg. Patients with a DBP ≤ 80 mmHg and NIHSS > 7 were more likely to be older (67.35 ± 14.95 vs. 62.92 ± 13.81) with higher rates of atrial fibrillation (19.8% vs. 7.4%), coronary artery disease (45.7% vs. 33.1%), history of drug or alcohol abuse (4.9% vs. 0.7%), dyslipidemia (58.0% vs. 51.4%), heart failure (16.0% vs. 6.1%), and hypertension (84.0% vs. 70.9%), but with lower rates of obesity (38.3% vs. 53.4%). This group significantly differed regarding ambulation status before admission, during admission, and at discharge. AIS patients with a DBP > 80 mmHg and NIHSS > 7 were more likely to be older (67.96 ± 14.67 vs. 61.95 ± 13.67) and less likely to be Caucasians (68.4% vs. 84.0%). In addition, they presented with higher rates of atrial fibrillation (17.7% vs. 6.9%), coronary artery disease (29.1% vs. 26.0%), heart failure (19.0% vs. 5.3%), hypertension (88.6% vs. 74.0%) and peripheral vascular disease (12.7% vs. 4.6%). They presented with higher INR (1.1 ± 0.27 vs. 1.01 ± 0.12), were less likely to be directly admitted for treatment (60.3% vs. 79.4%), and were more likely to show improvement in ambulation (60.9% vs. 41.2%). 

In the adjusted analysis of AIS patients with a DBP ≤ 80 mmHg, obesity (OR = 0.388, 95% Cl, 0.182–0.828, *p* = 0.014) was associated with reduced stroke severity, while an increased heart rate (OR = 1.025, 95% Cl, 1.001–1.050, *p* = 0.042) was associated with higher stroke severity (Table 3). The predictive power of the model was moderately strong with area under the curve (AUROC) = 0.670 (95% Cl, 0.593–0.746, *p* < 0.001). Table 4 presents the risk factors in AIS patients with a DBP > 80 mmHg. Hypertension (OR = 3.453, 95% Cl, 1.137–10.491, *p* = 0.029), history of smoking (OR = 2.55, 95% Cl, 1.06–6.132, *p* = 0.037), and heart rate (OR = 1.036, 95% Cl, 1.009–1.064, *p* = 0.009) were associated with a higher stroke severity. Caucasians (OR = 0.294, 95% Cl, 0.090–0.964, *p* = 0.002) and obesity (OR = 0.455, 95% Cl, 0.207–1.002, *p* = 0.05) were more likely to be associated with reduced stroke severity. The model’s predictive power was moderately strong, as shown by the AUROC, which is 0.644 (95% Cl, 0.568–0.720, *p* < 0.001).

## 4. Discussion

In this study, we characterized risk factors associated with stroke severities among AIS patients with DBP ≤ 80 mm Hg and >80 mmHg. Heart rate was associated with increased stroke severity in the adjusted analysis, while obesity was associated with reduced stroke severity in AIS patients with a DBP ≤ 80 mmHg. In addition, three potentially modifiable risk factors—hypertension, smoking history, and heart rate—were associated with higher stroke severity. In contrast, Caucasians and obesity were associated with reduced stroke severity in AIS patients with a DBP > 80 mmHg. 

In previous studies, lower BP levels were associated with improved prognosis in patients treated with AIS, while higher BP levels were associated with poor treatment outcomes [28,29]. Our results extend beyond previous reports on elevated BP and stroke severity in the non-telestroke setting [22,23,30,31]. Most studies did not report specific risk factors associated with stroke severity in AIS patients with elevated DBP.

The current study reveals that elevated heart rate was associated with increased stroke severity among AIS patients with a DBP ≤ 80 mmHg. This finding was also observed in AIS patients with DBP > 80 mmHg. Lower heart rates are directly linked with decreased mortality among patients with heart failure [32]. In this group, the optimal heart rate appeared to be between 70–76; for every 10-point increase in heart rate, the risk of poor outcomes increased by 10% [33]. While optimum heart rate differed among populations, low heart rates in AIS patients may lead to hypoperfusion in ischemic areas and adverse outcomes [34]. In addition, heart rate during the acute period of ischemic stroke is a predictor of major clinical events [35], and optimal heart rate control is always targeted to prevent subsequent cardiovascular events [36]. Patients with elevated heart rates present with different comorbid conditions, including infection, dehydration, hyperthyroidism, or arrhythmia, and these commodities contribute to heart rate elevation [37]. In addition, elevated heart rate is also reported to be a marker of elevated sympathetic activity due to stress response to stroke [38]. This causes pathophysiologic effects, such as induced oxidative stress and endothelial dysfunction, leading to atherosclerosis [39]. Ventricular dysfunction, caused by prolonged tachycardia, decreased coronary perfusion, and renal dysfunction, is a plausible explanation for the adverse outcomes caused by elevated heart rate [40,41]. These findings lend credence to our current result of an elevated heart rate associated with increased stroke severity. Moreover, a high heart rate can cause either hypoperfusion to ischemic brain regions, where cerebral autoregulation is diminished or absent, resulting in further brain damage and adverse outcomes [42]. Our study does not address whether lowering heart rate to a specific target would be beneficial in AIS with a DBP ≤ 80 mmHg. Future studies are necessary to determine the role of elevated heart rate in stroke severity in AIS patients with elevated DBP.

In the current study, obesity was associated with a reduced stroke severity in AIS patients with a DBP ≤ 80 mmHg. This finding was also observed for AIS patients with a DBP ≤ 80 mmHg. Despite obesity being an established risk factor for stroke, several studies reported a better outcome after stroke in obese and overweight patients, giving the impression of a survival advantage associated with obesity (i.e., the obesity stroke paradox). A gradient of increasing blood pressure with higher levels of BMI suggests that BMI may cause a direct effect on blood pressure, independent of other clinical risk factors. The description of obesity categories is based on BMI [43], and there is a U-shaped association between BMI and stroke [44,45]. While the independent effect of BMI on stroke severity can be estimated with a multivariate adjustment for differences in comorbid conditions [46], differences in stroke severities due to the effect of specific risk factors cannot be reliably adjusted mainly because the severity associated with individual risk factors is difficult to be quantified. In addition, the severity of stroke associated with the specific effect of each risk factor may not be adequately controlled. Therefore, our findings are not in line with the possibility that risk factors in obese patients with stroke are less severe than in patients of normal weight [47]. 

The mechanisms of the obesity paradox are not very clear. Several possible explanations have been proposed including a paracrine effect of adipose tissue [48]. Importantly, the number of obese patients with severe aortic stenosis scheduled for transcatheter aortic valve implantation (TAVI) is high and will continue to increase as a result of the aging of the population [49]. There is also an argument that the obese group, which consists of younger patients with potentially lower procedural risk and might contribute to the biased outcome. This is because younger patients may seek earlier medical care, and therefore, more aggressive treatment with cardioprotective medication could produce a beneficial outcome of interventional treatment [48,50]. Some studies have argued that BMI is an independent predictor of improved survival even after adjusting for the effects of age and gender. In support of this, many studies have reported that a population with increased BMI received more efficient medical care compared with those with a normal BMI [15]. Obese patients are reported to present with higher metabolic reserves, and this may benefit them in critical health conditions [51]. While an incremental role of metabolic reserves in the resistance of damaging effects of acute morbidities has been proposed [52], an obesity paradox has been reported among CAD patients, such that obese patients present with reduced all-cause mortality compared with patients with normal BMI [53]. In a meta-analysis study, overweight BMI was associated with reduced cardiac mortality; whereas obese BMI was not [54]. Moreover, obese patients presented with significantly reduced cardiovascular and non-cardiovascular mortality compared with those with normal BMI [54]. Reduced cardiovascular mortality was associated with several factors including the misclassification of lean and fat mass by BMI [55] more aggressive risk factor management in obese patients [56] and improved endothelial function [55,57]. The presence of unadjusted confounding factors, selection and lead time bias, and genetic differences are other factors that have been implicated in reduced cardiovascular mortality among overweight and obese patients.

We observed that AIS patients with a history of hypertension and elevated measured diastolic pressure after initial stroke were associated with increased stroke severity. Hypertension is associated with an increased risk of initial stroke and the control of hypertension reduces this risk [21,58,59]. Randomized control trials have continuously challenged the “lower, the better” hypothesis for hypertension [6]. The inconsistent results revealed by some of the clinical trials have led to unclear blood pressure treatment targets for AIS patients. Some studies indicated that elevated BP levels is a poor prognostic factor for AIS [60,61], while other studies [62,63] did not find any association. Therefore, managing hypertension in the acute stage of ischemic stroke remains controversial. Our results provide evidence that high DBP > 80 mmHg is associated with increased stroke severity in AIS patients with a history of hypertension. The association between hypertension and stroke severity is strong and direct [64]. As shown in the current study, stroke severity in AIS patients with elevated DBP > 80 mmHg is linked with AIS patients with a history of hypertension. Further prospective studies are needed to determine the specific effect of hypertension on stroke severity with the increase in DBP. 

We observed that AIS patients with a DBP > 80 mmHg and smoking history were associated with higher stroke severity. Several studies across various ethnicities and populations demonstrate a strong association between smoking and stroke risk [65,66]. Findings reveal that current smokers present at least a two-to-fourfold increased risk of stroke compared with lifelong nonsmokers or individuals who had quit smoking more than 10 years prior [65]. Stroke-related severity is reported to stem from tobacco smoke, which contains more than 3000 different chemicals that promote the development of free radicals, inducing vascular endothelial dysfunction and inflammation [67]. This ultimately leads to the development and acceleration of the atherosclerotic process [68]. Smoking also causes hypercoagulability, which is the increased tendency of blood to thrombose [65]. This causes an increase in fibrinogen concentration, a decrease in fibrinolytic activity, and an increase in the aggregation of platelets [69]. The consequence is a decrease in cerebral blood flow, which may further increase the risk of clot formation, subsequent stroke risk, and its severity through a slowed flow or stasis phenomenon [70].

We observed that Caucasians were associated with reduced stroke severity in AIS patients with a DBP > 80 mmHg. Stroke is a significant cause of long-term disability [71], and the burden and fatality of stroke are higher in racial/ethnic minorities [72]. Minority individuals are reported to present with more hypertension, diabetes, and obesity when compared with non-Hispanic whites [73]. In general, African Americans, Hispanics, and Native Americans have higher stroke risks, stroke occurrence at an earlier age, and for some minorities, possibly more severe strokes than non-Hispanic whites [74]. Our finding of reduced stroke severity in Caucasian AIS patients with DBP > 80 mmHg is supported by studies indicating that a higher prevalence of risk factors [75], lower socioeconomic status [76], and health care system challenges for minority patients [77] may contribute to higher stroke severity when compared with Caucasian AIS patients. Our results lend further credence to the suggestions that breaking down barriers to care is an important step to take critical steps toward reducing stroke disparities.

## 5. Limitations

There are also some limitations to this study that should be considered in interpreting the results. First, we do not have data on how BP measurements were taken. It is possible that BP levels were determined using several measurements. Therefore, there is the possibility of measurement errors that might result in underestimating or overestimating the association between DBP levels and stroke severity. This study included only inpatients in one regional telestroke network, resulting in small sample sizes, and thus the findings cannot be generalized. Since this is a retrospective data analysis approach, there is also possible selection bias due to lack of control or inability to quantify the severity of the individual risk factors.

## 6. Conclusions

In our findings, hypertension, chronic renal disease, and increased heart rate were associated with worsening neurologic functions in patients with DBP ≤ 80 mmHg. Obese AIS patients with DBP ≤ 80 mmHg presented with reduced stroke severity. Hypertension, smoking history, and increased heart rate were associated with increased stroke severity in patients with DBP > 80 mmHg. Obesity or Caucasian race in AIS patients with DBP > 80 mmHg were associated with reduced stroke severity. Therefore, this study identified different risk factors associated with stroke severities among AIS patients based on their DBP levels. The recognition of identified risk factors can help refine the prognosis and improve the care of AIS patients with elevated DBP. Further study is needed to learn more about DBP as an independent risk factor for stroke to possibly improve and expand treatment for AIS patients in the telestroke network.

## Figures and Tables

**Table 1:** Demographic and clinical characteristics of ischemic stroke patients divided by diastolic blood pressure ≤ 80 mmHg or >80 mmHg. Results for continuous variables are presented as mean ± SD, while discrete data are presented as percentage frequency. Pearson’s Chi-squared was used to compare demographic and clinical characteristics differences in patients with a diastolic blood pressure ≤ 80 mmHg or > 80 mmHg.

Characteristic	Diastolic Blood Pressure ≤ 80 mmHg	Diastolic Blood Pressure > 80 mmHg	
Number of Patients	239	213	*p*-value
Age Group: No. (%)			
<50	40 (16.7)	31 (14.6)	0.226
50–59	37 (15.5)	46 (21.6)	
60–69	62 (25.9)	59 (27.7)	
70–79	65 (27.2)	42 (19.7)	
≥80	35 (14.6)	35 (16.4)	
Mean ± SD	64.46 ± 14.51	64.36 ± 14.3	0.937
Race: No (%)			
White	203 (84.9)	167 (78.4)	0.109
Black	28 (11.7)	40 (18.8)	
Other	8 (3.3)	6 (2.8)	
Gender: No. (%)			
Female	134 (56.1)	98 (46.0)	0.033 *^a^
Male	105 (43.9)	115 (54.0)	
Hispanic Ethnicity: No. (%)	3 (1.3)	6 (2.8)	0.235
BMI: Mean ± SD	29.6 ± 7.05	29.61 ± 7.13	0.985
Medical History: No. (%)			
Atrial Fib	29 (12.1)	24 (11.3)	0.775
Coronary Artery Disease	89 (37.2)	58 (27.2)	0.023 *^a^
Carotid Artery Stenosis	10 (4.2)	11 (5.2)	0.621
Depression	32 (13.4)	28 (13.1)	0.939
Diabetes	93 (38.9)	83 (39.0)	0.990
Drugs or Alcohol	8 (3.3)	10 (4.7)	0.465
Dyslipidemia	128 (53.6)	102 (47.9)	0.229
Stroke Family History	29 (12.1)	22 (10.3)	0.545
Heart Failure	24 (10.0)	23 (10.8)	0.793
Hormonal Replacement Therapy	7 (2.9)	3 (1.4)	0.273
Hypertension	182 (76.2)	170 (79.8)	0.349
Migraine	6 (2.5)	6 (2.8)	0.840
Obesity	112 (46.9)	112 (52.6)	0.225
Previous Stroke	57 (23.8)	48 (22.5)	0.741
Previous TIA (>24 h)	25 (10.5)	23 (10.8)	0.907
Peripheral Vascular Disease	14 (5.9)	17 (8.0)	0.373
Chronic Renal Disease	10 (4.2)	10 (4.7)	0.792
Sleep Apnea	6 (2.5)	8 (3.8)	0.446
Smoker	66 (27.9)	62 (29.1)	0.725
Medication History: No (%)			
HTN Medication	162 (67.8)	149 (70.0)	0.619
Cholesterol Reducer	113 (47.3)	92 (43.2)	0.384
Diabetes Medication	72 (30.1)	63 (29.6)	0.899
Antidepressant	33 (13.8)	28 (13.1)	0.837
Initial NIHSS Score: No (%)			
0–9	168 (76.4)	143 (70.4)	0.505
10–14	21 (9.5)	27 (13.3)	
15–20	20 (9.1)	23 (11.3)	
21–25	11 (5.0)	10 (4.9)	
Mean ± SD	7.39 ± 8.1	7.8 ± 7.32	0.587
Lab values: Mean ± SD			
Total cholesterol	167.02 ± 41.62	171.11 ± 46.21	0.336
Triglycerides	145.96 ± 91.79	143.44 ± 99.38	0.785
HDL	39.75 ± 11.81	40.33 ± 12.69	0.621
LDL	101.63 ± 33.96	105.58 ± 38.6	0.259
Lipids	6.42 ± 1.59	6.5 ± 1.97	0.651
Blood Glucose	137.2 ± 72.02	142.32 ± 80.6	0.483
Serum Creatinine	1.06 ± 0.52	1.14 ± 1.02	0.311
INR	1.07 ± 0.24	1.05 ± 0.2	0.300
Vital Signs: Mean ± SD			
Heart Rate	75.62 ± 15.25	81.43 ± 15.99	<0.001 *^b^
Blood Pressure Systolic	137.24 ± 22.92	157.33 ± 22.1	<0.001 *^b^
Ambulation Status Prior to Event: No. (%)			
Ambulate Independently	226 (94.6)	204 (95.8)	0.850
Ambulate with Assistance	4 (1.7)	2 (0.9)	
Unable to Ambulate	4 (1.7)	4 (1.9)	
Not Documented	5 (2.1)	3 (1.4)	
Ambulation Status on Admission: No. (%)			
Ambulate Independently	60 (25.1)	55 (25.8)	0.424
Ambulate with Assistance	67 (28.0)	46 (21.6)	
Unable to Ambulate	55 (23.0)	58 (27.2)	
Not Documented	57 (23.8)	54 (25.4)	
Ambulation Status on Discharge: No. (%)			
Ambulate Independently	128 (53.6)	111 (52.1)	0.874
Ambulate with Assistance	67 (28.0)	62 (29.1)	
Unable to Ambulate	31 (13.0)	25 (11.7)	
Not Documented	13 (5.4)	15 (7.0)	
rtPA Received: No. (%)	159 (66.5)	146 (68.5)	0.648
Emergency Department	66 (27.7)	59 (27.8)	0.981
Direct Admission	172 (72.3)	153 (72.2)	
Improved Ambulation: No. (%)	109 (47.8)	94 (47.5)	0.945
NIHSS > 7: No. (%)	81 (35.4)	79 (37.6)	0.625

Notes: ^a^ Pearson’s Chi-squared test. ^b^ Student’s *t*-test. * *p*-value < 0.05.

**Table 2:** Demographic and clinical characteristics of an NIHSS score > 7 in ischemic stroke patients in the telestroke network stratified by diastolic blood pressure ≤ 80 mmHg or >80 mmHg. Results for continuous variables are presented as mean ± SD, while discrete data are presented as percentage frequency. Pearson’s Chi-squared is used to compare differences between demographic and clinical characteristics in groups with an NIHSS score greater than 7 in the telestroke based on diastolic blood pressure ≤ 80 mmHg or >80 mmHg.

	Diastolic Blood Pressure ≤ 80 mmHg			Diastolic Blood Pressure ≥ 80 mmHg		
Characteristic	NIHSS ≤ 7	NIHSS > 7		NIHSS ≤ 7	NIHSS > 7	
Number of Patients	148	81	*p*-value	131	79	*p*-Value
Age Group: No. (%)						
<50 years	26 (17.6)	12 (14.8)	0.007 *^a^	25 (19.1)	6 (7.6)	0.010 *^a^
50–59	24 (16.2)	10 (12.3)		27 (20.6)	19 (24.1)	
60–69	41 (27.7)	21 (25.9)		42 (32.1)	16 (20.3)	
70–79	45 (30.4)	17 (21.0)		22 (16.8)	19 (24.1)	
≥80	12 (8.1)	21 (25.9)		15 (11.5)	19 (24.1)	
Age Mean ± SD	62.92 ± 13.81	67.35 ± 14.95	0.025 *^b^	61.95 ± 13.67	67.96 ± 14.67	0.003 *^b^
Race: No (%)						
White	129 (87.2)	66 (81.5)	0.474	110 (84.0)	54 (68.4)	0.009 *^a^
Black	15 (10.1)	11 (13.6)		20 (15.3)	20 (25.3)	
Other	4 (2.7)	4 (4.9)		1 (0.8)	5 (6.3)	
Gender: No. (%)						
Female	89 (60.1)	41 (50.6)	0.165	56 (42.7)	40 (50.6)	0.267
Male	59 (39.9)	40 (49.4)		75 (57.3)	39 (49.4)	
Hispanic Ethnicity: No. (%)	3 (2.0)	0 (0.0)	0.197	2 (1.5)	4 (5.1)	0.136
BMI: Mean ± SD	29.89 ± 6.52	29.36 ± 8.02	0.595	29.71 ± 6.55	29.32 ± 8.14	0.703
Medical History: No. (%)						
Atrial Fib	11 (7.4)	16 (19.8)	0.006 *^a^	9 (6.9)	14 (17.7)	0.015 *^a^
Coronary Artery Disease	49 (33.1)	37 (45.7)	0.060	34 (26.0)	23 (29.1)	0.618
Carotid Artery Stenosis	6 (4.1)	4 (4.9)	0.754	7 (5.3)	4 (5.1)	0.930
Depression	20 (13.5)	11 (13.6)	0.989	17 (13.0)	11 (13.9)	0.845
Diabetes	59 (39.9)	31 (38.3)	0.813	49 (37.4)	32 (40.5)	0.655
Drugs or Alcohol	1 (0.7)	4 (4.9)	0.035 *^a^	7 (5.3)	3 (3.8)	0.610
Dyslipidemia	76 (51.4)	47 (58.0)	0.333	63 (48.1)	37 (46.8)	0.860
Stroke Family History	20 (13.5)	8 (9.9)	0.422	11 (8.4)	11 (13.9)	0.205
Heart Failure	9 (6.1)	13 (16.0)	0.014 *^a^	7 (5.3)	15 (19.0)	0.002 *^a^
Hormonal Replacement Therapy	5 (3.4)	2 (2.5)	0.702	2 (1.5)	1 (1.3)	0.877
Hypertension	105 (70.9)	68 (84.0)	0.029 *^a^	97 (74.0)	70 (88.6)	0.011 *^a^
Migraine	6 (4.1)	0 (0.0)	0.066	5 (3.8)	1 (1.3)	0.282
Obesity	79 (53.4)	31 (38.3)	0.029 *^a^	73 (55.7)	38 (48.1)	0.284
Previous Stroke	32 (21.6)	20 (24.7)	0.596	28 (21.4)	19 (24.1)	0.652
Previous TIA (>24 h)	15 (10.1)	9 (11.1)	0.818	13 (9.9)	9 (11.4)	0.736
Peripheral Vascular Disease	9 (6.1)	5 (6.2)	0.978	6 (4.6)	10 (12.7)	0.033 *^a^
Chronic Renal Disease	4 (2.7)	6 (7.4)	0.096	7 (5.3)	3 (3.8)	0.610
Sleep Apnea	5 (3.4)	1 (1.2)	0.332	6 (4.6)	2 (2.5)	0.453
Smoker	42 (28.4)	21 (25.9)	0.691	42 (32.1)	20 (25.3)	0.299
Medication History: No (%)						
HTN Medication	93 (62.8)	61 (75.3)	0.055	86 (65.6)	60 (75.9)	0.116
Cholesterol Reducer	74 (50.0)	35 (43.2)	0.325	60 (45.8)	30 (38.0)	0.267
Diabetes Medication	43 (29.1)	28 (34.6)	0.388	39 (29.8)	22 (27.8)	0.766
Antidepressant	21 (14.2)	11 (13.6)	0.899	18 (13.7)	10 (12.7)	0.823
Lab Values: Mean ± SD						
Total cholesterol	167.41 ± 41.51	165.64 ± 42.15	0.764	169.97 ± 47.2	172.57 ± 44.77	0.700
Triglycerides	150.96 ± 85.73	136.38 ± 102.82	0.265	146.77 ± 85.48	136.38 ± 120.92	0.474
HDL	38.98 ± 11.83	40.87 ± 11.21	0.252	39.32 ± 12.11	42.22 ± 13.52	0.117
LDL	102.42 ± 34.15	100.01 ± 33.95	0.619	104.87 ± 39.73	106.53 ± 36.72	0.769
Lipids	6.33 ± 1.45	6.63 ± 1.82	0.186	6.48 ± 1.95	6.52 ± 2.04	0.896
Blood Glucose	134.03 ± 64.81	147.01 ± 86.22	0.210	137.71 ± 76.53	150.24 ± 87.97	0.284
Serum Creatinine	1.04 ± 0.56	1.07 ± 0.41	0.769	1.12 ± 0.99	1.18 ± 1.09	0.709
INR	1.04 ± 0.15	1.09 ± 0.31	0.154	1.01 ± 0.12	1.1 ± 0.27	0.019 *^b^
Vital Signs: Mean ± SD						
Heart Rate	74.11 ± 12.69	77.8 ± 18.4	0.111	79.79 ± 14.72	84.49 ± 17.75	0.050
Blood Pressure Systolic	136.22 ± 22.26	140.42 ± 24.23	0.188	156.84 ± 22.32	158.8 ± 21.85	0.536
Blood Pressure Diastolic	68.09 ± 8.84	67.11 ± 8.17	0.409	94.12 ± 11.9	93.52 ± 11.74	0.721
Ambulation Status Prior to Event: No. (%)						
Ambulate Independently	145 (98.0)	72 (88.9)	0.014 *^a^	129 (98.5)	74 (93.7)	0.200
Ambulate with Assistance	0 (0.0)	3 (3.7)		1 (0.8)	1 (1.3)	
Unable to Ambulate	2 (1.4)	2 (2.5)		1 (0.8)	2 (2.5)	
Not Documented	1 (0.7)	4 (4.9)		0 (0.0)	2 (2.5)	
Ambulation Status on Admission: No. (%)						
Ambulate Independently	52 (35.1)	5 (6.2)	<0.001 *^a^	52 (39.7)	3 (3.8)	<0.001 *^a^
Ambulate with Assistance	50 (33.8)	15 (18.5)		36 (27.5)	9 (11.4)	
Unable to Ambulate	5 (3.4)	46 (56.8)		8 (6.1)	48 (60.8)	
Not Documented	41 (27.7)	15 (18.5)		35 (26.7)	19 (24.1)	
Ambulation Status on Discharge: No. (%)						
Ambulate Independently	109 (73.6)	15 (18.5)	<0.001 *^a^	89 (67.9)	21 (26.6)	<0.001 *^a^
Ambulate with Assistance	31 (20.9)	34 (42.0)		38 (29.0)	24 (30.4)	
Unable to Ambulate	5 (3.4)	24 (29.6)		4 (3.1)	19 (24.1)	
Not Documented	3 (2.0)	8 (9.9)		0 (0.0)	15 (19.0)	
rtPA Administration	100 (67.6)	56 (69.1)	0.808	89 (67.9)	57 (72.2)	0.521
Emergency Department	40 (27.2)	23 (28.4)	0.848	27 (20.6)	31 (39.7)	0.003 *^a^
Direct Admission	107 (72.8)	58 (71.6)		104 (79.4)	47 (60.3)	
Improved Ambulation: No (%)	71 (49.0)	37 (49.3)	0.959	54 (41.2)	39 (60.9)	0.010 *^a^

Notes: ^a^ Pearson’s Chi-squared test. ^b^ Student’s *t*-test. * *p*-value < 0.05.

**Table 3:** Clinical factors associated with stroke severity in AIS patients with DBP ≤ 80 mmHg in the telestroke network. Adjusted OR < 1 denotes factors associated with not having an NIHSS score > 7, while OR > 1 denotes factors associated with having an NIHSS score > 7. Hosmer–Lemeshow test (*p* = 0.318), Cox and Snell (*R*^2^ = 0.100). The overall classified percentage of 68.1% was applied to check for the fitness of the logistic regression model. * Indicates statistical significance (*p* < 0.05) with a 95% confidence interval. Classification table (overall correctly classified percentage = 68.1%) and area under the ROC curve (AUC = 0.670, 0.593–0.746) were applied to check model fitness.

				95% C.I.		
Variables	B Value	Wald	Odds Ratio	Lower	Upper	*p*-Value
Hypertension	0.817	2.813	2.263	0.871	5.876	0.094
Obesity	−0.946	6.001	0.388	0.182	0.828	0.014 *
Chronic Renal Disease	1.748	3.546	5.746	0.931	35.456	0.06
Heart Rate	0.025	4.118	1.025	1.001	1.05	0.042 *

**Table 4:** Clinical factors associated with an NIHSS score > 7 for ischemic stroke patients with a diastolic blood pressure > 80 mmHg in the telestroke network. Adjusted OR < 1 denotes factors associated with not having an NIHSS score > 7, while OR > 1 denotes factors associated with having an NIHSS score > 7. Hosmer–Lemeshow test (*p* = 0.820), Cox and Snell (*R*^2^ = 0.142). The overall classified percentage of 70.6% was applied to check for the fitness of the logistic regression model. * Indicates statistical significance (*p* < 0.05) with a 95% confidence interval. Classification table (overall correctly classified percentage = 70.6%) and area under the ROC curve (AUC = 0.644, 0.568–0.720) were applied to check model fitness.

				95% C.I.		
Variables	B Value	Wald	Odds Ratio	Lower	Upper	*p*-Value
Caucasian	−1.223	4.085	0.294	0.09	0.964	0.043 *
Hypertension	1.239	4.778	3.453	1.137	10.491	0.029 *
Obesity	−0.787	3.825	0.455	0.207	1.002	0.05
History of Smoking	0.936	4.369	2.55	1.06	6.132	0.037 *
Heart rate	0.035	6.887	1.036	1.009	1.064	0.009 *

## Data Availability

The data presented in this study are available on request from the corresponding author.
